# Robust-stein estimator for overcoming outliers and multicollinearity

**DOI:** 10.1038/s41598-023-36053-z

**Published:** 2023-06-05

**Authors:** Adewale F. Lukman, Rasha A. Farghali, B. M. Golam Kibria, Okunlola A. Oluyemi

**Affiliations:** 1 Department of Epidemiology and Biostatistics, University of Medical Sciences, Ondo, Nigeria; 2 Department of Mathematics, University of Medical Sciences, Ondo, Nigeria; 3 Department of Mathematics, Insurance and Applied Statistics, Helwan University, Cairo, Egypt; 4 Department of Mathematics and Statistics, Florida International University, Miami, USA

**Keywords:** Environmental sciences, Mathematics and computing

## Abstract

Linear regression models with correlated regressors can negatively impact the performance of ordinary least squares estimators. The Stein and ridge estimators have been proposed as alternative techniques to improve estimation accuracy. However, both methods are non-robust to outliers. In previous studies, the M-estimator has been used in combination with the ridge estimator to address both correlated regressors and outliers. In this paper, we introduce the robust Stein estimator to address both issues simultaneously. Our simulation and application results demonstrate that the proposed technique performs favorably compared to existing methods.

## Introduction

Linear regression models are popularly adopted to predict the response variable from a combination of regressors or predictors. The model is generally written as:

1.1 $$y=X\beta +\varepsilon ,$$

where *y* is an $n\times 1$ vector of response variable, $X$ is a $n\times p$ full rank matrix of regressors, $\beta$ is a $p\times 1$ vector of unknown regression coefficients, $\varepsilon$ is an $n\times 1$ vector of errors. The error term is assumed to be normally distributed with mean zero and constant variance ${\sigma }^{2}{I}_{n},$ ${I}_{n}$ is an $n\times n$ identity matrix. The parameter $\beta$ is often estimated using the ordinary least squares estimator (OLS) which is defined as follows:

1.2 $$\widehat{\beta }={\left({X}^{^{\prime}}X\right)}^{-1}{X}^{^{\prime}}y$$

1.3 $$Cov(\widehat{\beta })={\sigma }^{2}({X}^{^{\prime}}X{)}^{-1}$$

where,${\widehat{\sigma }}^{2}$ is the estimated residual mean square, ${\widehat{\sigma }}^{2}=\frac{{\left(Y-X\widehat{\beta }\right)}^{^{\prime}} \left(Y-X\widehat{\beta }\right)}{n-{p}^{*}}$. The scalar mean squared error $(\mathrm{S}MSE)$ of $\widehat{\beta }$ and the matrix mean squared error $(\mathrm{M}MSE)$ of $\widehat{\beta }$ are calculated as:

1.4 $$MMSE\left(\widehat{\beta }\right)={\sigma }^{2}({X}^{^{\prime}}X{)}^{-1}$$

1.5 $$SMSE\left(\widehat{\beta }\right)={\sigma }^{2}tr({X}^{^{\prime}}X{)}^{-1}={\sigma }^{2}\sum_{j=1}^{{p}^{*}}\frac{1}{{\lambda }_{j}}$$

is known to be sensitive to the presence of correlated regressors (multicollinearity) and outliers, which can negatively impact its performance. Several alternative methods have been proposed to address the issue of correlated regressors, including the Stein estimator, ridge regression, Liu estimator, Modified Liu estimator, modified ridge-type estimator, Kibra-Lukman estimator, Dawoud-Kibria estimator, and others^1–7^. These methods aim to effectively account for the correlation among the regressors.

Outliers are data points that differ significantly from other observations and can have a substantial impact on model estimates^8,9^. They threatened the efficiency of the OLS estimator^8–11^, and it is well-known that robust estimators are preferred when dealing with outliers^12–19^. However, both multicollinearity and outliers can exist simultaneously in a model. To address both issues, some of the methods mentioned earlier have been combined. For example, ridge regression has been combined with the M-estimator to handle both correlated regressors and outliers in the y-direction^20^.

Recently, the Stein estimator has gained popularity as an alternative to OLS and performs well in handling correlated regressors. Few researchers have extended the method to some generalized linear models such as the Poisson, the zero-inflated negative binomial and inverse gaussian regression models^21–23^. However, it is sensitive to outliers in the y-direction. In this study, we propose a robust version of the Stein estimator that can handle both multicollinearity and outliers.

In Section “Theoretical comparisons among estimators”, we provide a theoretical comparison of the proposed and existing estimators. We then conduct a simulation study in Section “Simulation study” to evaluate their performance, and in Section “Real-life application”, we analyze real-life data for illustration purposes. Finally, we conclude our findings in Section “Some concluding remarks”.

## Theoretical comparisons among estimators

With the suggested biased estimators, we employ the spectral decomposition of the information matrix ($X^{\prime } X$) to offer the explicit form of the matrix mean squared error (MMSE) and the scalar mean squared error (SMSE). Assume that there exists a matrix $T$ such that:

$$T\left({X}^{^{\prime}}X\right){T}^{\mathrm{^{\prime}}}=\Lambda =diag\left\{{\lambda }_{j}\right\} , j=\mathrm{1,2},\dots ,{p}^{*},\left({p}^{*}=p+1\right),$$

where, ${\lambda }_{1}\ge {\lambda }_{2} \ge ..\ge {\lambda }_{{p}^{*}}$, are the ordered eigenvalues of $({X}^{^{\prime}}X)$ and $T$ is a $({p}^{*}\times {p}^{*})$ orthogonal matrix whose columns are the corresponding eigenvectors of ${\lambda }_{1}\ge {\lambda }_{2} \ge ..\ge {\lambda }_{{p}^{*}}$. Rewrite the linear regression model in Eq. (1.1) in canonical form:

2.1 $${y}_{i}={\sum }_{j=1}^{{p}^{*}}{\alpha }_{j}{h}_{ij}+{\varepsilon }_{i}, i=\mathrm{1,2},\dots ,n,$$

where $\text{H}\,=\,\acute{{XT}}, \alpha =\acute{T\beta }, T\left(X{^{\prime}}X\right){T}{^{\prime}}={H}{^{\prime}}H=\Lambda$. With the presence of correlated regressors (multicollinearity) the ordinary least squares estimator ${\widehat{\alpha }}_{OLS}$ is inadequate and inefficient. Also, outlier(s) negatively affect the parameter estimates of ${\widehat{\alpha }}_{LS}$. The M-estimator is efficient for handling outliers in the y-direction^15^. Let ${\widehat{\alpha }}_{M}$ be the M-estimator of α, and can be obtained across a solution of M-estimating equations. The effects of outliers in the y-direction are eliminated by the weights of the residuals in the iterative reweighted least-squares approach used to solve M-estimating equations^10,15^.

2.2 $${\widehat{\alpha }}_{LS}={\Lambda }^{-1}{H}^{^{\prime}}y$$

2.3 $${\widehat{\alpha }}_{M }=min\sum_{i=1}^{n}\pi \left(\frac{{\varepsilon }_{i}}{\eta }\right)=min\sum_{i=1}^{n}\pi \left(\frac{{y}_{i}-{\sum }_{j=1}^{{p}^{*}}{\alpha }_{j}{h}_{ij}}{\eta }\right),$$

where $\pi (.)$ indicates a robust criterion function and $\eta$ is a scale parameter estimate. ${\widehat{\alpha }}_{M}$ is obtained through a solution of M-estimating equations $\sum_{i=1}^{n}\phi \left(\frac{{e}_{i}}{\eta }\right)=0$ and $\sum_{i=1}^{n}\phi \left(\frac{{e}_{i}}{\eta }\right){x}_{i}=0$, where, ${e}_{i}={y}_{i}-{\sum }_{j=1}^{{p}^{*}}{\widehat{\alpha }}_{j-M}{h}_{ij} , \phi ={\pi }^{^{\prime}}$ is a useful selected function^10^.

2.4 $$SMSE\left({\widehat{\alpha }}_{M}\right)={\sum }_{j=1}^{{p}^{*}}{\Psi }_{jj},$$

where ${\Psi }_{jj}$ is the ${j}$th element of the main diagonal of the matrix $Var\left({\widehat{\alpha }}_{M}\right)=\Psi$, which is finite.

The ridge regression estimator of $\alpha$ is defined as:

2.5 $${{\widehat{\alpha }}_{Ridge }=\left(\Lambda +kI\right)}^{-1}\Lambda {\widehat{\alpha }}_{LS}$$

2.6 $$cov\left({\widehat{\alpha }}_{Ridge }\right)={\sigma }^{2}{\left(\Lambda +kI\right)}^{-1}\Lambda {\left(\Lambda +kI\right)}^{-1}, k\ge 0$$

$$Bias\left({\widehat{\alpha }}_{Ridge }\right)=E\left({\left(\Lambda +kI\right)}^{-1}\Lambda {\widehat{\alpha }}_{LS }\right)-\alpha$$

2.7 $$=\left[{\left(\Lambda +kI\right)}^{-1}\Lambda -I\right]\beta$$

The scalar mean squared error $(\mathrm{S}MSE)$ of ${\widehat{\alpha }}_{Ridge}$ and the matrix mean squared error $(\mathrm{M}MSE)$ of ${\widehat{\alpha }}_{Ridge}$ are calculated as:

2.8 $$MMSE\left({\widehat{\alpha }}_{Ridge }\right)={\sigma }^{2}{\left(\Lambda +kI\right)}^{-1}\Lambda {\left(\Lambda +kI\right)}^{-1}+Bias\left({\widehat{\alpha }}_{Ridge }\right)Bias\left({\widehat{\alpha }}_{Ridge }\right){^{\prime}}$$

2.9 $$SMSE\left({\widehat{\alpha }}_{Ridge }\right)={\sigma }^{2}\sum_{j=1}^{{p}^{*}}\frac{{\lambda }_{j}}{{\left({\lambda }_{j}+{k}\right)}^{2}}+{k}^{2}\sum_{j=1}^{{p}^{*}}\frac{{\alpha }_{j}^{2}}{{\left({\lambda }_{j}+{k}\right)}^{2}}$$

The M-Ridge is given by:

2.10 $${\widehat{\alpha }}_{M-ridge}={\left(\Lambda +{k}_{m}I\right)}^{-1}\Lambda {\widehat{\alpha }}_{M}$$

2.11 $$cov\left({\widehat{\alpha }}_{Ridge-M }\right)={\left(\Lambda +{k}_{m}I\right)}^{-1}\mathrm{\Lambda \psi \Lambda }{\left(\Lambda +{k}_{m}I\right)}^{-1}, k\ge 0$$

2.12 $$Bias\left({\widehat{\alpha }}_{Ridge-M }\right)=E\left({\left(\Lambda +{k}_{m}I\right)}^{-1}\Lambda {\widehat{\alpha }}_{M }\right)-\alpha$$

The scalar mean squared error $(\mathrm{S}MSE)$ of ${\widehat{\alpha }}_{Ridge-M}$ and the matrix mean squared error $(\mathrm{M}MSE)$ of ${\widehat{\alpha }}_{Ridge-M}$ are calculated as:

2.13 $$MMSE\left({\widehat{\alpha }}_{Ridge-M }\right)={\left(\Lambda +{k}_{m}I\right)}^{-1}\mathrm{\Lambda \Psi \Lambda }{\left(\Lambda +{k}_{m}I\right)}^{-1}+Bias\left({\widehat{\alpha }}_{Ridge-M }\right)Bias\left({\widehat{\alpha }}_{Ridge-M }\right){^{\prime}}$$

2.14 $$SMSE\left({\widehat{\alpha }}_{Ridge-M }\right)=\sum_{j=1}^{p*}\frac{{\lambda }_{j}^{2}}{{\left({\lambda }_{j}+k\right)}^{2}}{\Psi }_{jj}+\sum_{j=1}^{p*}\frac{{\alpha }_{j}^{2}{k}^{2}}{{\left({\lambda }_{j}+k\right)}^{2}}$$

The James–Stein estimator (Stein, 1960) is given by:

2.15 $${\widehat{\alpha }}_{JSE }=\mathrm{c }{\widehat{\alpha }}_{LS}$$

where

2.16 $$c=\frac{{\widehat{\alpha }}_{LS }{^{\prime}}{\widehat{\alpha }}_{LS }}{{\widehat{\alpha }}_{LS }{^{\prime}}{\widehat{\alpha }}_{LS }+{\sigma }^{2}tr(X{^{\prime}}X{)}^{-1})}={\sum }_{j=1}^{{p}^{*}}\frac{{\lambda }_{j}{\alpha }_{j}^{2}}{{\sigma }^{2}+{\lambda }_{j}{\alpha }_{j}^{2}}$$

2.17 $$cov\left({\widehat{\alpha }}_{JSE }\right)=c Cov\left({\widehat{\alpha }}_{JSE }\right){c}^{^{\prime}}=c {\widehat{\sigma }}^{2}(X{^{\prime}}X{)}^{-1}c{^{\prime}}$$

$$Bias\left({\widehat{\alpha }}_{JSE }\right)=E\left(c{\widehat{\alpha }}_{JSE }\right)-\alpha =(c-1)\alpha$$

The scalar mean squared error $(\mathrm{S}MSE)$ of ${\widehat{\alpha }}_{JSE}$ and the matrix mean squared error $(\mathrm{M}MSE)$ of ${\widehat{\alpha }}_{JSE}$ are calculated as:

2.18 $$MMSE\left({\widehat{\alpha }}_{JSE }\right)=c {\sigma }^{2}({X}^{^{\prime}}X{)}^{-1}{c}^{^{\prime}}+Bias\left({\widehat{\alpha }}_{JSE }\right)Bias\left({\widehat{\alpha }}_{JSE }\right){^{\prime}}$$

2.19 $$SMSE\left({\widehat{\alpha }}_{JSE }\right)={c}^{2}{\sigma }^{2}\sum_{j=1}^{{p}^{*}}\frac{1}{{\lambda }_{j}}+{\left(c-1\right)}^{2}\sum_{j=1}^{{p}^{*}}{\alpha }_{j}^{2}$$

2.20 $$SMSE\left({\widehat{\alpha }}_{JSE}\right)={\sum }_{j=1}^{{p}^{*}}\frac{{\sigma }^{2}{\lambda }_{j}{\alpha }_{j}^{4}}{{\left({\sigma }^{2}+{\lambda }_{j}{\alpha }_{j}^{2}\right)}^{2}}+{\sum }_{j=1}^{{p}^{*}}\frac{{\sigma }^{4}{\alpha }_{j}^{2}}{{\left({\sigma }^{2}+{\lambda }_{j}{\alpha }_{j}^{2}\right)}^{2}}$$

### M-Stein estimator

Stein estimator is sensitive to outliers in the y-direction. Thus, there is a need to propose the Robust Stein estimator which is defined as follows:

2.21 $${\widehat{\alpha }}_{M-JSE}={c}^{*} {\widehat{\alpha }}_{M },$$

where ${\widehat{\alpha }}_{M}$ is the M-estimate of α,

2.22 $$ {c}^{*}={\sum }_{j=1}^{{p}^{*}}\left(\frac{{\lambda }_{j}{\alpha }_{M}^{2}}{{\Psi }_{jj}+{\lambda }_{j}{\alpha }_{M}^{2}}\right)$$

2.23 $$cov\left({\widehat{\alpha }}_{M-JSE }\right)={c}^{*}Cov\left({\widehat{\alpha }}_{M }\right){{c}^{*}}^{^{\prime}}={c}^{*}\uppsi (X{^{\prime}}X{)}^{-1}{c}^{*}{^{\prime}}$$

$$Bias\left({\widehat{\alpha }}_{M-JSE }\right)=E\left({c}^{*}{\widehat{\alpha }}_{M }\right)-\alpha =({c}^{*}-1)\alpha$$

The scalar mean squared error $(\mathrm{S}MSE)$ of ${\widehat{\alpha }}_{M-JSE}$ and the matrix mean squared error $(\mathrm{M}MSE)$ of ${\widehat{\alpha }}_{M-JSE}$ are calculated as:

2.24 $$MMSE\left({\widehat{\alpha }}_{M-JSE }\right)={c}^{*}\uppsi ({X}^{^{\prime}}X{)}^{-1}{{c}^{*}}^{^{\prime}}+Bias\left({\widehat{\alpha }}_{M }\right)Bias\left({\widehat{\alpha }}_{M }\right){^{\prime}}$$

2.25 $$SMSE\left({\widehat{\alpha }}_{M-JSE }\right)={{c}^{*}}^{2}\Psi \sum_{j=1}^{{p}^{*}}\frac{1}{{\lambda }_{j}}+{\left({c}^{*}-1\right)}^{2}\sum_{j=1}^{{p}^{*}}{\alpha }_{j}^{2}$$

2.26 $$SMSE\left({\widehat{\alpha }}_{M-JSE}\right)={\sum }_{j=1}^{{p}^{*}}\frac{{\Psi }_{jj}{\lambda }_{j}{\alpha }_{j}^{4}}{{\left({\Psi }_{jj}+{\lambda }_{j}{\alpha }_{j}^{2}\right)}^{2}}+{\sum }_{j=1}^{{p}^{*}}\frac{{\Psi }_{jj}^{2}{\alpha }_{j}^{2}}{{\left({\Psi }_{jj}+{\lambda }_{j}{\alpha }_{j}^{2}\right)}^{2}}$$

We presume the following conditions hold to describe the major theorems:

1. $\phi$ is skew-symmetric and non-decreasing.
2. The errors are symmetric.
3. $$\Psi$$ is finite.

Now we will give the theoretical comparisons among the estimators based on the scalar mean squared errors, presented in Eqs. (1.5), (2.4), (2.9), (2.14), and (2.20).

#### Theorem 2.1

$SMSE\left({\widehat{\alpha }}_{M-JSE}\right)<SMSE\left({\widehat{\alpha }}_{LS}\right),$ if ${\sigma }^{2}\left({\Psi }_{jj}+{\lambda }_{j}{\alpha }_{j}^{2}\right)>{\Psi }_{jj}{\alpha }_{j}^{2}{\lambda }_{j}$, where ${\Psi }_{jj}$ is the ${j}$th element of the main diagonal of the matrix $Var\left({\widehat{\alpha }}_{M}\right)=\Psi$.

#### Proof:

The difference between $SMSE\left({\widehat{\alpha }}_{M-JSE}\right) \mathrm{and} SMSE\left({\widehat{\alpha }}_{LS}\right)$ is given by:

2.27 $${\sum }_{j=1}^{{p}^{*}}\frac{{\Psi }_{jj}{\lambda }_{j}{\alpha }_{j}^{4}}{{\left({\Psi }_{jj}+{\lambda }_{j}{\alpha }_{j}^{2}\right)}^{2}}+{\sum }_{j=1}^{{p}^{*}}\frac{{\Psi }_{jj}^{2}{\alpha }_{j}^{2}}{{\left({\Psi }_{jj}+{\lambda }_{j}{\alpha }_{j}^{2}\right)}^{2}}-{\sum }_{j=1}^{{p}^{*}}\frac{{\sigma }^{2}}{{\lambda }_{j}}$$

$${\sum }_{j=1}^{{p}^{*}}\frac{{\Psi }_{jj}{\lambda }_{j}^{2}{\alpha }_{j}^{4}+{\Psi }_{jj}^{2}{\lambda }_{j}{\alpha }_{j}^{2}-{\sigma }^{2}{\left({\Psi }_{jj}+{\lambda }_{j}{\alpha }_{j}^{2}\right)}^{2}}{{\lambda }_{j}{\left({\Psi }_{jj}+{\lambda }_{j}{\alpha }_{j}^{2}\right)}^{2}}<0$$

$$\left({\Psi }_{jj}+{\lambda }_{j}{\alpha }_{j}^{2}\right){\Psi }_{jj}{\alpha }_{j}^{2}{\lambda }_{j}<{\sigma }^{2}{\left({\Psi }_{jj}+{\lambda }_{j}{\alpha }_{j}^{2}\right)}^{2}$$

$${\Psi }_{jj}{\alpha }_{j}^{2}{\lambda }_{j}<{\sigma }^{2}\left({\Psi }_{jj}+{\lambda }_{j}{\alpha }_{j}^{2}\right)$$

2.28 $${\Psi }_{jj}{\alpha }_{j}^{2}{\lambda }_{j}-{\sigma }^{2}\left({\Psi }_{jj}+{\lambda }_{j}{\alpha }_{j}^{2}\right)<0$$

It is obvious from Eq. (2.28) that ${\sigma }^{2}\left({\Psi }_{jj}+{\lambda }_{j}{\alpha }_{j}^{2}\right)$ is greater than ${\Psi }_{jj}{\alpha }_{j}^{2}{\lambda }_{j}$. Thus, the difference is less than zero and the proof is completed.

#### Theorem 2.2

$SMSE\left({\widehat{\alpha }}_{M-JSE}\right)<SMSE\left({\widehat{\alpha }}_{Ridge}\right),$ if ${\sigma }^{2}{\lambda }_{j}\left({\alpha }_{j}^{2}{\lambda }_{j}+{\Psi }_{jj}\right)+{k}^{2}{\alpha }_{j}^{4}{\lambda }_{j}>{\Psi }_{jj}{\alpha }_{j}^{2}{\lambda }_{j}\left({\lambda }_{j}+2k\right)$, where ${\Psi }_{jj}$ is the ${j}^{th}$ element of the main diagonal of the matrix $Var\left({\widehat{\alpha }}_{M}\right)=\Psi$.

#### Proof:

The difference between $SMSE\left({\widehat{\alpha }}_{M-JSE}\right) and SMSE\left({\widehat{\alpha }}_{Ridge}\right)$ is given by:

2.29 $${\sum }_{j=1}^{{p}^{*}}\frac{{\Psi }_{jj}{\lambda }_{j}{\alpha }_{j}^{4}}{{\left({\Psi }_{jj}+{\lambda }_{j}{\alpha }_{j}^{2}\right)}^{2}}+{\sum }_{j=1}^{{p}^{*}}\frac{{\Psi }_{jj}^{2}{\alpha }_{j}^{2}}{{\left({\Psi }_{jj}+{\lambda }_{j}{\alpha }_{j}^{2}\right)}^{2}}-{\sum }_{j=1}^{{p}^{*}}\frac{\left({\sigma }^{2}{\lambda }_{j}+{k}^{2}{\alpha }_{j}^{2}\right)}{{\left({\lambda }_{j}+k\right)}^{2}}$$

$${\sum }_{j=1}^{{p}^{*}}\frac{{\Psi }_{jj}{\alpha }_{j}^{2}\left({\lambda }_{j}{\alpha }_{j}^{2}+{\Psi }_{jj}\right){\left({\lambda }_{j}+k\right)}^{2}-\left({\sigma }^{2}{\lambda }_{j}+{k}^{2}{\alpha }_{j}^{2}\right){\left({\Psi }_{jj}+{\lambda }_{j}{\alpha }_{j}^{2}\right)}^{2}}{{\left({\Psi }_{jj}+{\lambda }_{j}{\alpha }_{j}^{2}\right)}^{2}{\left({\lambda }_{j}+k\right)}^{2}}$$

$SMSE\left({\widehat{\alpha }}_{M-JSE}\right)$ is better than $SMSE\left({\widehat{\alpha }}_{Ridge}\right)$ if the difference is less than zero, i.e. if

$${\Psi }_{jj}{\alpha }_{j}^{2}\left({\lambda }_{j}{\alpha }_{j}^{2}+{\Psi }_{jj}\right){\left({\lambda }_{j}+k\right)}^{2}<\left({\sigma }^{2}{\lambda }_{j}+{k}^{2}{\alpha }_{j}^{2}\right){\left({\Psi }_{jj}+{\lambda }_{j}{\alpha }_{j}^{2}\right)}^{2}$$

$${\Psi }_{jj}{\alpha }_{j}^{2}{\left({\lambda }_{j}+k\right)}^{2}<\left({\sigma }^{2}{\lambda }_{j}+{k}^{2}{\alpha }_{j}^{2}\right)\left({\lambda }_{j}{\alpha }_{j}^{2}+{\Psi }_{jj}\right)$$

$${\Psi }_{jj}{\alpha }_{j}^{2}{\lambda }_{j}^{2}+{\Psi }_{jj}{\alpha }_{j}^{2}{k}^{2}+2k{\lambda }_{j}{\Psi }_{jj}{\alpha }_{j}^{2}<{\sigma }^{2}{\alpha }_{j}^{2}{\lambda }_{j}^{2}+{\sigma }^{2}{\lambda }_{j}{\Psi }_{jj}+{k}^{2}{\alpha }_{j}^{4}{\lambda }_{j}+{k}^{2}{\alpha }_{j}^{2}{\Psi }_{jj}$$

$${\Psi }_{jj}{\alpha }_{j}^{2}{\lambda }_{j}^{2}-2k{\lambda }_{j}{\Psi }_{jj}{\alpha }_{j}^{2}-{\sigma }^{2}{\alpha }_{j}^{2}{\lambda }_{j}^{2}+{\sigma }^{2}{\lambda }_{j}{\Psi }_{jj}+{k}^{2}{\alpha }_{j}^{4}{\lambda }_{j}< 0$$

2.30 $${\Psi }_{jj}{\alpha }_{j}^{2}{\lambda }_{j}\left({\lambda }_{j}+2k\right)-{\sigma }^{2}{\lambda }_{j}\left({\alpha }_{j}^{2}{\lambda }_{j}+{\Psi }_{jj}\right)-{k}^{2}{\alpha }_{j}^{4}{\lambda }_{j}<0$$

It is obvious from Eq. (2.30) that ${\sigma }^{2}{\lambda }_{j}\left({\alpha }_{j}^{2}{\lambda }_{j}+{\Psi }_{jj}\right)+{k}^{2}{\alpha }_{j}^{4}{\lambda }_{j}$ is greater than ${\Psi }_{jj}{\alpha }_{j}^{2}{\lambda }_{j}\left({\lambda }_{j}+2k\right)$. Thus, the difference is less than zero and the proof is completed.

#### Theorem 2.3

$SMSE\left({\widehat{\alpha }}_{M-JSE}\right)<SMSE\left({\widehat{\alpha }}_{M-Ridge}\right),$ if $\left({\Psi }_{jj}^{2}{\lambda }_{j}+{k}^{2}{\alpha }_{j}^{2}{\Psi }_{jj}+{k}^{2}{\alpha }_{j}^{4}{\lambda }_{j}\right)>{\Psi }_{jj}{\alpha }_{j}^{2}k\left(k+2{\lambda }_{j}\right)$, where ${\Psi }_{jj}$ is the ${j}^{th}$ element of the main diagonal of the matrix $Var\left({\widehat{\alpha }}_{M}\right)=\Psi$.

#### Proof:

The difference between $SMSE\left({\widehat{\alpha }}_{M-JSE}\right) and SMSE\left({\widehat{\alpha }}_{M-Ridge}\right)$ is given by:

2.31 $${\sum }_{j=1}^{{p}^{*}}\frac{{\Psi }_{jj}{\lambda }_{j}{\alpha }_{j}^{4}}{{\left({\Psi }_{jj}+{\lambda }_{j}{\alpha }_{j}^{2}\right)}^{2}}+{\sum }_{j=1}^{{p}^{*}}\frac{{\Psi }_{jj}^{2}{\alpha }_{j}^{2}}{{\left({\Psi }_{jj}+{\lambda }_{j}{\alpha }_{j}^{2}\right)}^{2}}-{\sum }_{j=1}^{{p}^{*}}\frac{{\Psi }_{jj}{\lambda }_{j}}{{\left({\lambda }_{j}+k\right)}^{2}}+{\sum }_{j=1}^{{p}^{*}}\frac{{k}^{2}{\alpha }_{j}^{2}}{{\left({\lambda }_{j}+k\right)}^{2}}$$

$${\sum }_{j=1}^{{p}^{*}}\frac{{\Psi }_{jj}{\alpha }_{j}^{2}\left({\lambda }_{j}{\alpha }_{j}^{2}+{\Psi }_{jj}\right)}{{\left({\Psi }_{jj}+{\lambda }_{j}{\alpha }_{j}^{2}\right)}^{2}}-{\sum }_{j=1}^{{p}^{*}}\frac{{\Psi }_{jj}{\lambda }_{j}+{k}^{2}{\alpha }_{j}^{2}}{{\left({\lambda }_{j}+k\right)}^{2}}$$

$${\sum }_{j=1}^{{p}^{*}}\frac{{\Psi }_{jj}{\alpha }_{j}^{2}\left({\lambda }_{j}{\alpha }_{j}^{2}+{\Psi }_{jj}\right){\left({\lambda }_{j}+k\right)}^{2}-\left({\Psi }_{jj}{\lambda }_{j}+{k}^{2}{\alpha }_{j}^{2}\right){\left({\Psi }_{jj}+{\lambda }_{j}{\alpha }_{j}^{2}\right)}^{2}}{{\left({\Psi }_{jj}+{\lambda }_{j}{\alpha }_{j}^{2}\right)}^{2}{\left({\lambda }_{j}+k\right)}^{2}}$$

$SMSE\left({\widehat{\alpha }}_{M-JSE}\right)$ is better than $SMSE\left({\widehat{\alpha }}_{M-Ridge}\right)$ if the difference is less than zero, i.e. if

$${\Psi }_{jj}{\alpha }_{j}^{2}\left({\lambda }_{j}{\alpha }_{j}^{2}+{\Psi }_{jj}\right){\left({\lambda }_{j}+k\right)}^{2}<\left({\Psi }_{jj}{\lambda }_{j}+{k}^{2}{\alpha }_{j}^{2}\right){\left({\Psi }_{jj}+{\lambda }_{j}{\alpha }_{j}^{2}\right)}^{2}$$

$${\Psi }_{jj}{\alpha }_{j}^{2}{\left({\lambda }_{j}+k\right)}^{2}<\left({\Psi }_{jj}{\lambda }_{j}+{k}^{2}{\alpha }_{j}^{2}\right)\left({\lambda }_{j}{\alpha }_{j}^{2}+{\Psi }_{jj}\right)$$

$${\Psi }_{jj}{\alpha }_{j}^{2}{k}^{2}+{\Psi }_{jj}{\alpha }_{j}^{2}{\lambda }_{j}^{2}+2{\Psi }_{jj}{\alpha }_{j}^{2}{\lambda }_{j}k<{\Psi }_{jj}{\alpha }_{j}^{2}{\lambda }_{j}^{2}+{\Psi }_{jj}^{2}{\lambda }_{j}+{k}^{2}{\alpha }_{j}^{2}{\Psi }_{jj}+{k}^{2}{\alpha }_{j}^{4}{\lambda }_{j}$$

2.32 $${\Psi }_{jj}{\alpha }_{j}^{2}k\left(k+2{\lambda }_{j}\right)-\left({\Psi }_{jj}^{2}{\lambda }_{j}+{k}^{2}{\alpha }_{j}^{2}{\Psi }_{jj}+{k}^{2}{\alpha }_{j}^{4}{\lambda }_{j}\right)<0$$

It is obvious from Eq. (2.32) that $\left({\Psi }_{jj}^{2}{\lambda }_{j}+{k}^{2}{\alpha }_{j}^{2}{\Psi }_{jj}+{k}^{2}{\alpha }_{j}^{4}{\lambda }_{j}\right)$ is greater than ${\Psi }_{jj}{\alpha }_{j}^{2}k\left(k+2{\lambda }_{j}\right)$. Thus, the difference is less than zero and the proof is completed.

#### Theorem 2.4

$SMSE\left({\widehat{\alpha }}_{M-JSE}\right)<SMSE\left({\widehat{\alpha }}_{JSE}\right),$ if ${\sigma }^{2}{\alpha }_{j}^{2}\left({\lambda }_{j}{\alpha }_{j}^{2}+{\Psi }_{jj}\right)>{\Psi }_{jj}{\alpha }_{j}^{2}\left({\lambda }_{j}{\alpha }_{j}^{2}+{\sigma }^{2}\right)$, where ${\Psi }_{jj}$ is the ${j}^{th}$ element of the main diagonal of the matrix $Var\left({\widehat{\alpha }}_{M}\right)=\Psi$.

#### Proof:

The difference between $SMSE\left({\widehat{\alpha }}_{M-JSE}\right) and SMSE\left({\widehat{\alpha }}_{JSE}\right)$ is given by:

2.33 $${\sum }_{j=1}^{{p}^{*}}\frac{{\Psi }_{jj}{\lambda }_{j}{\alpha }_{j}^{4}}{{\left({\Psi }_{jj}+{\lambda }_{j}{\alpha }_{j}^{2}\right)}^{2}}+{\sum }_{j=1}^{{p}^{*}}\frac{{\Psi }_{jj}^{2}{\alpha }_{j}^{2}}{{\left({\Psi }_{jj}+{\lambda }_{j}{\alpha }_{j}^{2}\right)}^{2}}-{\sum }_{j=1}^{{p}^{*}}\frac{{\sigma }^{2}{\lambda }_{j}{\alpha }_{j}^{4}}{{\left({\sigma }^{2}+{\lambda }_{j}{\alpha }_{j}^{2}\right)}^{2}}+{\sum }_{j=1}^{{p}^{*}}\frac{{\sigma }^{4}{\alpha }_{j}^{2}}{{\left({\sigma }^{2}+{\lambda }_{j}{\alpha }_{j}^{2}\right)}^{2}}$$

$${\sum }_{j=1}^{{p}^{*}}\frac{{\Psi }_{jj}{\alpha }_{j}^{2}\left({\lambda }_{j}{\alpha }_{j}^{2}+{\Psi }_{jj}\right){\left({\sigma }^{2}+{\lambda }_{j}{\alpha }_{j}^{2}\right)}^{2}-{\sigma }^{2}{\alpha }_{j}^{2}\left({\lambda }_{j}{\alpha }_{j}^{2}+{\sigma }^{2}\right){\left({\Psi }_{jj}+{\lambda }_{j}{\alpha }_{j}^{2}\right)}^{2}}{{\left({\Psi }_{jj}+{\lambda }_{j}{\alpha }_{j}^{2}\right)}^{2}{\left({\sigma }^{2}+{\lambda }_{j}{\alpha }_{j}^{2}\right)}^{2}}$$

$SMSE\left({\widehat{\alpha }}_{M-JSE}\right)$ is better than $SMSE\left({\widehat{\alpha }}_{JSE}\right)$ if the difference is less than zero, i.e. if

$${\Psi }_{jj}{\alpha }_{j}^{2}\left({\lambda }_{j}{\alpha }_{j}^{2}+{\sigma }^{2}\right)<{\sigma }^{2}{\alpha }_{j}^{2}\left({\lambda }_{j}{\alpha }_{j}^{2}+{\Psi }_{jj}\right)$$

2.34 $${\Psi }_{jj}{\alpha }_{j}^{2}\left({\lambda }_{j}{\alpha }_{j}^{2}+{\sigma }^{2}\right)-{\sigma }^{2}{\alpha }_{j}^{2}\left({\lambda }_{j}{\alpha }_{j}^{2}+{\Psi }_{jj}\right)<0$$

It is obvious from Eq. (2.34) that ${\sigma }^{2}{\alpha }_{j}^{2}\left({\lambda }_{j}{\alpha }_{j}^{2}+{\Psi }_{jj}\right)$ is greater than ${\Psi }_{jj}{\alpha }_{j}^{2}\left({\lambda }_{j}{\alpha }_{j}^{2}+{\sigma }^{2}\right)$. Thus, the difference is less than zero and the proof is completed.

## Simulation study

This section provides a simulation study using the R programming language to compare the performance of the non-robust and robust estimators.

### Simulation design

The design of this simulation study is based on specifying the variables that are anticipated to have an impact on the features of suggested estimator and selecting a metric to assess the outcomes. Following the cited references^24–28^, we generated the regressors as follows:

3.1 $${x}_{ij}={(1-{\rho }^{2})}^{1/2}{m}_{ij}+\rho {m}_{i,{p}^{*}+1}, i=\mathrm{1,2},\dots ,n, j=\mathrm{1,2},3, \dots , {p}^{*}$$

where ${m}_{ij}$ independent standard normal are pseudo-random numbers, ${p}^{*}$ denotes the number of regressors (${p}^{*}$=4,8,12) and $\rho$ denotes level of multicollinearity ($\rho =\mathrm{0.7,0.8}, 0.9, 0.99)$. Thus, the response variable is given by:

3.2 $${y}_{i}={\beta }_{0}+{\beta }_{1}{x}_{i1}+ \dots + {\beta }_{{p}^{*}}{x}_{i{p}^{*}}+{\varepsilon }_{i}, i=\mathrm{1,2}, \dots , n$$

where ${\varepsilon }_{i}\sim N(0,{\sigma }^{2})$, $\sigma =\mathrm{5,10}$, n = 30,50,100, 200 and the regression parameters are chosen such that ${\beta }^{^{\prime}}\beta =1$^29–35^. The experiment is repeated 2000 times. We introduced outlier by increasing the magnitude of the response variable. Using Eq. (3.3), 10% and 20% contamination were added to the model.

3.3 $${y}_{i}=h*\mathrm{max}\left({y}_{i}\right)+{y}_{i},$$

where *h* = 10 is added to inflate the response variable^36,37^. The ridge parameter *k* is obtained using the following equation:

3.4 $$k=\frac{{p}^{*}{\widehat{\sigma }}^{2}}{\sum_{j=1}^{{p}^{*}}{\alpha }_{LS}^{2}},$$

where ${\widehat{\sigma }}^{2}=\frac{\sum_{j=1}^{n}{e}_{i}^{2}}{n-r}$, ${e}_{i}=y-\widehat{y}$ and *r* denotes the number of estimated parameter.

The unbiased estimator of ${\Psi }_{jj}$ is asymptotically ${\widehat{A}}^{2}{\lambda }_{j}^{-1}$ where ${\widehat{A}}^{2}={s}^{2}{(n-{p}^{*})}^{-1}$ $\frac{\sum_{i=1}^{n}{\left[\varphi \left({e}_{i}/s\right)\right]}^{2}}{\sum_{i=1}^{n}{\left[\frac{1}{n}{\varphi }^{^{\prime}}\left({e}_{i}/s\right)\right]}^{2}} \mathrm{and }s\mathrm{ is the scale estimate}.$ Thus, the parameter for M-Ridge is determined using the following equation:

3.5 $${k}_{m}=\frac{{p}^{*}\widehat{A}}{\sum_{j=1}^{{p}^{*}}{\alpha }_{M}^{2}}$$

The estimated mean squared error (MSE) is computed as follows:

3.6 $$MSE= \frac{1}{2000} \sum_{i=1}^{2000}\sum_{j=1}^{p}{({\widehat{\beta }}_{ij}-{\beta }_{j})}^{2}$$

where, ${\widehat{\beta }}_{ij}$ is the estimated ${j}^{th}$ parameter in the ${i}^{th}$ replication and ${\beta }_{j}$ is the ${j}^{th}$ true parameter value. The estimated values of the mean squared error (MSE) of the proposed and other estimators are displayed in Tables 1, 2, 3, 4, 5 and 6 for ${p}^{*}$=4 with 10% outliers, ${p}^{*}$=8 with 10% outliers, ${p}^{*}$=12 with 10% outliers, ${p}^{*}$=4 with 20% outliers, ${p}^{*}$=8 with 20% outliers and ${p}^{*}$=12 with 10% outliers respectively.

**Table 1:** Estimated MSE values for p = 4 with 10% outlier.

n	30		50		100		200	
$\sigma$	5	10	5	10	5	10	5	10
$$\rho =0.7$$								
${\widehat{\alpha }}_{LS}$	334.39	1269.12	161.47	603.63	93.26	351.43	63.83	240.57
${\widehat{\alpha }}_{Ridge}$	96.85	366.83	46.70	173.68	27.25	102.11	17.91	66.91
${\widehat{\alpha }}_{JSE}$	184.43	700.17	64.68	240.06	36.35	135.85	27.73	103.58
${\widehat{\alpha }}_{M}$	5.32	20.56	2.10	7.65	1.04	3.50	0.63	2.02
${\widehat{\alpha }}_{M-JSE}$	1.44	3.33	0.94	1.09	0.92	0.96	0.90	0.95
$$\rho =0.8$$								
${\widehat{\alpha }}_{LS}$	448.39	1687.28	225.57	832.85	123.56	460.96	91.91	343.37
${\widehat{\alpha }}_{Ridge}$	126.51	475.31	63.19	232.75	35.01	130.31	24.90	92.68
${\widehat{\alpha }}_{JSE}$	276.00	1039.44	105.17	386.62	54.55	202.55	46.33	172.56
${\widehat{\alpha }}_{M}$	6.81	26.60	2.70	10.16	1.24	4.34	0.96	2.68
${\widehat{\alpha }}_{M-JSE}$	2.00	5.83	0.99	1.37	0.92	1.00	0.90	0.98
$$\rho =0.9$$								
${\widehat{\alpha }}_{LS}$	800.34	2977.67	427.24	1556.29	217.49	800.67	178.59	660.46
${\widehat{\alpha }}_{Ridge}$	219.03	814.35	114.90	418.77	59.15	217.92	46.51	172.06
${\widehat{\alpha }}_{JSE}$	569.86	2122.70	246.97	898.20	117.37	431.58	109.51	405.46
${\widehat{\alpha }}_{M}$	11.51	45.45	4.69	18.17	1.91	7.05	1.33	4.79
${\widehat{\alpha }}_{M-JSE}$	4.65	17.39	1.37	3.17	0.97	1.28	0.94	1.25
$$\rho =0.99$$								
${\widehat{\alpha }}_{LS}$	1511.38	5579.67	4162.02	14,948.83	1916.26	6939.70	1753.83	6423.20
${\widehat{\alpha }}_{Ridge}$	406.91	1501.78	1068.82	3847.27	493.89	1794.36	438.01	1609.34
${\widehat{\alpha }}_{JSE}$	1189.76	4395.97	3499.75	12,571.39	1557.44	5642.43	1494.22	5480.46
${\widehat{\alpha }}_{M}$	21.02	83.54	41.94	167.18	14.36	56.87	10.98	43.39
${\widehat{\alpha }}_{M-JSE}$	12.76	51.89	38.09	159.73	9.08	36.91	8.88	35.62

**Table 2:** Estimated MSE values for p = 8 with 10% outlier.

n	30		50		100		200	
$\sigma$	5	10	5	10	5	10	5	10
$$\rho =0.7$$								
${\widehat{\alpha }}_{LS}$	991.49	3547.59	367.15	1333.23	286.72	1020.75	156.87	562.54
${\widehat{\alpha }}_{Ridge}$	237.09	849.14	92.03	333.94	70.85	251.95	38.94	139.05
${\widehat{\alpha }}_{JSE}$	574.11	2053.58	135.14	490.23	124.55	442.87	63.65	227.09
${\widehat{\alpha }}_{M}$	16.54	65.49	4.61	17.71	2.46	9.18	1.19	4.16
${\widehat{\alpha }}_{M-JSE}$	3.43	12.54	0.984	1.24	0.954	1.13	0.928	0.974
$$\rho =0.8$$								
${\widehat{\alpha }}_{LS}$	1409.59	4924.47	495.44	1758.07	429.76	1498.04	231.65	812.34
${\widehat{\alpha }}_{Ridge}$	334.20	1169.31	121.90	432.56	104.61	364.68	56.54	197.75
${\widehat{\alpha }}_{JSE}$	887.78	3103.49	205.21	728.04	215.46	751.23	108.97	381.09
${\widehat{\alpha }}_{M}$	22.79	90.53	5.87	22.79	3.44	13.11	1.61	5.79
${\widehat{\alpha }}_{M-JSE}$	5.93	24.07	1.04	1.57	1.01	1.49	0.931	1.05
$$\rho =0.9$$								
${\widehat{\alpha }}_{LS}$	2656.92	9036.34	887.95	3059.53	886.05	2953.05	460.32	1575.24
${\widehat{\alpha }}_{Ridge}$	625.83	2132.56	214.23	739.22	207.93	709.76	110.58	378.01
${\widehat{\alpha }}_{JSE}$	1867.80	6360.33	448.73	1546.77	522.67	1784.46	264.46	904.02
${\widehat{\alpha }}_{M}$	41.69	166.13	9.82	38.64	6.46	25.17	2.86	10.80
${\widehat{\alpha }}_{M-JSE}$	16.40	71.37	1.41	3.58	1.45	3.84	1.02	1.69
$$\rho =0.99$$								
${\widehat{\alpha }}_{LS}$	24,559.35	81,192.05	7951.18	26,482.60	8764.16	29,262.26	4611.21	15,395.13
${\widehat{\alpha }}_{Ridge}$	5778.64	19,129.54	1888.04	6305.34	2080.10	6959.70	1092.61	3649.50
${\widehat{\alpha }}_{JSE}$	21,413.86	70,814.03	6231.93	20,727.48	7366.58	24,626.64	3803.09	12,689.94
${\widehat{\alpha }}_{M}$	413.20	1759.63	81.87	326.48	61.04	243.37	25.63	101.84
${\widehat{\alpha }}_{M-JSE}$	377.51	1509.54	46.05	206.76	45.81	202.69	18.04	79.30

**Table 3:** Estimated MSE values for p = 12 with 10% outlier.

n	30		50		100		200	
$\sigma$	5	10	5	10	5	10	5	10
$$\rho =0.7$$								
${\widehat{\alpha }}_{LS}$	2604.94	8829.07	896.69	3051.38	460.96	1534.91	218.28	770.90
${\widehat{\alpha }}_{Ridge}$	543.53	1839.01	207.90	705.95	108.26	359.45	52.83	186.11
${\widehat{\alpha }}_{JSE}$	1640.62	5557.62	411.12	1396.73	186.55	618.17	84.12	296.52
${\widehat{\alpha }}_{M}$	67.04	267.59	12.19	48.06	3.67	13.96	1.52	5.42
${\widehat{\alpha }}_{M-JSE}$	21.63	96.12	1.53	4.07	0.965	1.14	0.937	0.971
$$\rho =0.8$$								
${\widehat{\alpha }}_{LS}$	3998.75	13,189.59	1339.33	4417.40	692.69	2225.49	309.65	1062.11
${\widehat{\alpha }}_{Ridge}$	828.70	2730.26	308.39	1014.78	161.00	516.32	74.14	253.85
${\widehat{\alpha }}_{JSE}$	2709.64	8935.37	696.87	2296.04	323.37	1035.55	138.39	474.24
${\widehat{\alpha }}_{M}$	98.46	393.05	17.225	68.20	5.10	19.70	1.97	7.23
${\widehat{\alpha }}_{M-JSE}$	38.60	174.10	2.28	7.97	1.01	1.52	0.937	1.02
$$\rho =0.9$$								
${\widehat{\alpha }}_{LS}$	8195.87	26,305.95	2686.94	8583.41	1400.06	4340.77	590.43	1953.99
${\widehat{\alpha }}_{Ridge}$	1686.83	5411.15	614.78	1959.70	322.48	999.12	140.00	462.84
${\widehat{\alpha }}_{JSE}$	6112.39	19,612.72	1662.91	5310.64	798.83	2473.15	325.43	1076.14
${\widehat{\alpha }}_{M}$	193.17	771.48	32.60	129.71	9.52	37.38	3.37	12.81
${\widehat{\alpha }}_{M-JSE}$	103.81	467.14	6.13	27.16	1.44	4.09	0.989	1.43
$$\rho =0.99$$								
${\widehat{\alpha }}_{LS}$	83,344.68	260,825.92	27,061.36	84,006.10	14,253.48	42,889.05	5726.77	18,177.61
${\widehat{\alpha }}_{Ridge}$	16,956.42	53,080.29	6146.78	19,047.96	3260.76	9814.84	1347.36	4276.90
${\widehat{\alpha }}_{JSE}$	74,897.53	234,279.84	22,940.79	71,219.34	11,801.17	35,511.63	4651.08	14,761.87
${\widehat{\alpha }}_{M}$	2188.69	9287.41	310.45	1241.09	90.29	360.41	28.81	114.52
${\widehat{\alpha }}_{M-JSE}$	1886.15	7542.64	246.94	1104.34	57.14	271.60	14.09	65.28

**Table 4:** Estimated MSE values for p = 4 with 20% outlier.

n	30		50		100		200	
$\sigma$	5	10	5	10	5	10	5	10
$$\rho =0.7$$								
${\widehat{\alpha }}_{LS}$	568.65	2141.13	293.37	1091.45	168.92	636.72	116.32	435.23
${\widehat{\alpha }}_{Ridge}$	138.77	520.48	79.29	293.44	45.92	172.49	30.47	113.22
${\widehat{\alpha }}_{JSE}$	288.18	1081.39	112.26	415.11	62.39	233.65	47.35	175.88
${\widehat{\alpha }}_{M}$	11.32	44.30	3.51	13.26	1.65	5.91	0.893	3.01
${\widehat{\alpha }}_{M-JSE}$	2.75	8.54	0.996	1.24	0.942	0.982	0.935	0.966
$$\rho =0.8$$								
${\widehat{\alpha }}_{LS}$	761.63	2842.37	411.19	1508.89	223.95	834.83	167.76	621.08
${\widehat{\alpha }}_{Ridge}$	180.69	672.05	107.77	393.73	59.09	219.85	42.53	156.83
${\widehat{\alpha }}_{JSE}$	439.51	1636.31	185.09	676.19	94.64	351.27	80.30	296.07
${\widehat{\alpha }}_{M}$	14.44	56.75	4.59	17.69	2.01	7.42	1.14	4.03
${\widehat{\alpha }}_{M-JSE}$	4.06	14.24	1.08	1.68	0.946	1.02	0.934	0.997
$$\rho =0.9$$								
${\widehat{\alpha }}_{LS}$	1358.01	5011.71	781.63	2825.33	394.51	1450.39	326.54	1194.91
${\widehat{\alpha }}_{Ridge}$	311.67	1146.83	197.02	709.15	99.85	366.71	79.70	291.15
${\widehat{\alpha }}_{JSE}$	929.02	3423.97	442.20	1593.99	206.74	758.25	193.35	706.26
${\widehat{\alpha }}_{M}$	24.34	96.18	8.10	31.78	3.20	12.22	1.95	7.27
${\widehat{\alpha }}_{M-JSE}$	9.41	37.10	1.65	4.32	0.999	1.34	0.976	1.28
$$\rho =0.99$$								
${\widehat{\alpha }}_{LS}$	10,198.91	12,876.85	7639.70	27,208.70	3484.80	12,593.20	3216.32	11,634.61
${\widehat{\alpha }}_{Ridge}$	2706.58	9834.81	1839.22	6520.47	835.04	3012.58	752.92	2724.06
${\widehat{\alpha }}_{JSE}$	10,876.85	39,605.41	6374.79	22,685.65	2820.07	10,189.28	2700.45	9761.21
${\widehat{\alpha }}_{M}$	267.48	1082.27	73.65	294.02	25.15	100.03	16.82	66.73
${\widehat{\alpha }}_{M-JSE}$	232.32	915.01	55.70	236.04	12.51	51.92	10.57	43.34

**Table 5:** Estimated MSE values for p = 8 with 20% outlier.

n	30		50		100		200	
$\sigma$	5	10	5	10	5	10	5	10
$$\rho =0.7$$								
${\widehat{\alpha }}_{LS}$	1680.29	6032.57	649.14	2347.64	519.84	1855.91	277.44	985.56
${\widehat{\alpha }}_{Ridge}$	338.58	1211.14	146.89	530.40	117.08	418.83	61.52	217.87
${\widehat{\alpha }}_{JSE}$	892.25	3197.52	220.07	795.30	215.90	771.14	107.57	380.48
${\widehat{\alpha }}_{M}$	77.05	295.95	9.04	35.45	4.09	15.66	1.73	6.28
${\widehat{\alpha }}_{M-JSE}$	50.56	191.36	1.15	1.97	0.975	1.16	0.949	0.986
$$\rho =0.8$$								
${\widehat{\alpha }}_{LS}$	2386.06	8366.73	876.71	3095.33	779.41	2726.55	410.10	1423.33
${\widehat{\alpha }}_{Ridge}$	474.54	1657.42	194.86	687.38	172.99	606.78	89.31	309.44
${\widehat{\alpha }}_{JSE}$	1404.79	4918.57	338.48	1195.82	377.11	1319.87	187.49	649.08
${\widehat{\alpha }}_{M}$	107.42	406.95	11.64	45.89	5.79	22.48	2.36	8.79
${\widehat{\alpha }}_{M-JSE}$	77.90	292.56	1.35	2.96	1.04	1.60	0.953	1.05
$$\rho =0.9$$								
${\widehat{\alpha }}_{LS}$	4497.41	15,343.62	1573.26	5386.61	1570.99	5376.51	815.20	2761.00
${\widehat{\alpha }}_{Ridge}$	884.37	3004.26	343.22	1174.22	343.98	1180.84	174.46	590.72
${\widehat{\alpha }}_{JSE}$	3030.80	10,326.90	755.01	2589.95	923.26	3161.07	462.72	1565.51
${\widehat{\alpha }}_{M}$	203.07	767.82	19.8	77.64	11.01	43.35	4.29	16.50
${\widehat{\alpha }}_{M-JSE}$	177.83	680.59	2.20	7.30	1.61	4.58	1.05	1.69
$$\rho =0.99$$								
${\widehat{\alpha }}_{LS}$	41,563.66	137,956.01	14,127.25	46,680.78	15,899.19	53,246.48	8168.09	26,990.81
${\widehat{\alpha }}_{Ridge}$	8117.10	26,834.85	3032.17	10,016.38	3442.46	11,567.05	1722.93	5695.03
${\widehat{\alpha }}_{JSE}$	36,040.86	119,596.46	10,875.79	35,998.75	13,146.46	44,050.06	6718.96	22,180.61
${\widehat{\alpha }}_{M}$	2663.99	10,224.36	164.43	657.03	105.31	420.56	39.30	156.52
${\widehat{\alpha }}_{M-JSE}$	1932.41	7299.72	86.21	387.18	66.60	297.11	21.73	97.40

**Table 6:** Estimated MSE values for p = 12 with 20% outlier.

n	30		50		100		200	
$\sigma$	5	10	5	10	5	10	5	10
$$\rho =0.7$$								
${\widehat{\alpha }}_{LS}$	4625.49	15,569.99	1592.00	5431.56	819.56	2736.17	384.88	1358.69
${\widehat{\alpha }}_{Ridge}$	795.44	2678.80	320.33	1093.17	171.78	574.02	81.58	287.60
${\widehat{\alpha }}_{JSE}$	2666.28	8958.67	657.99	2242.06	311.30	1040.14	135.03	475.13
${\widehat{\alpha }}_{M}$	704.82	2441.50	37.13	144.53	6.41	24.91	2.40	8.90
${\widehat{\alpha }}_{M-JSE}$	620.10	2178.46	10.24	37.24	0.987	1.21	0.955	0.987
$$\rho =0.8$$								
${\widehat{\alpha }}_{LS}$	1358.69	23,312.47	2375.22	7861.05	1231.96	3968.68	545.98	1872.88
${\widehat{\alpha }}_{Ridge}$	1217.42	3984.70	474.01	1570.14	255.44	823.97	114.40	392.03
${\widehat{\alpha }}_{JSE}$	4490.75	14,662.86	1130.21	3735.51	547.12	1764.94	225.07	770.29
${\widehat{\alpha }}_{M}$	1173.73	3987.40	53.34	206.79	8.99	35.23	3.16	11.93
${\widehat{\alpha }}_{M-JSE}$	938.69	3239.22	17.50	65.90	1.06	1.71	0.958	1.04
$$\rho =0.9$$								
${\widehat{\alpha }}_{LS}$	14,664.60	46,636.56	4759.12	15,267.63	2492.38	7745.98	1039.84	3447.79
${\widehat{\alpha }}_{Ridge}$	2489.93	7922.75	942.43	3028.36	512.12	1594.44	215.61	714.32
${\widehat{\alpha }}_{JSE}$	10,386.80	32,979.34	2750.90	8815.89	1376.18	4285.06	537.64	1780.84
${\widehat{\alpha }}_{M}$	2697.90	8905.19	103.67	398.83	16.94	67.04	5.50	21.27
${\widehat{\alpha }}_{M-JSE}$	1894.77	6371.29	46.18	179.03	1.67	5.36	1.02	1.51
$$\rho =0.99$$								
${\widehat{\alpha }}_{LS}$	150,036.15	464,788.19	47,914.59	149,338.60	25,396.00	76,608.56	10,075.28	32,095.55
${\widehat{\alpha }}_{Ridge}$	25,236.70	78,230.32	9413.00	29,398.07	5186.20	15,674.68	2073.34	6598.45
${\widehat{\alpha }}_{JSE}$	132,438.10	410,155.40	39,657.19	123,665.60	20,860.67	62,998.27	7982.77	25,401.38
${\widehat{\alpha }}_{M}$	34,454.37	112,577.69	1015.75	3886.20	162.04	647.37	48.07	191.56
${\widehat{\alpha }}_{M-JSE}$	18,785.63	61,944.42	1004.59	4039.57	88.94	425.72	17.89	84.86

**Figure 1:**
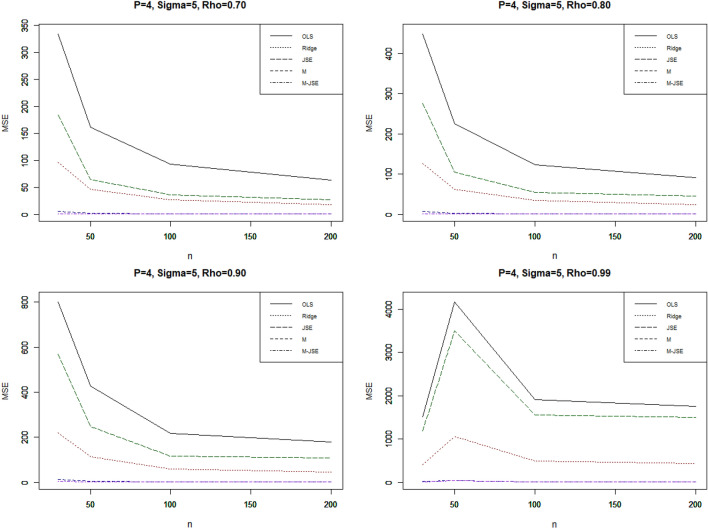
MSE vs sample size**,** For p = 4, σ = 5, 10% outliers and different ρ.

**Figure 2:**
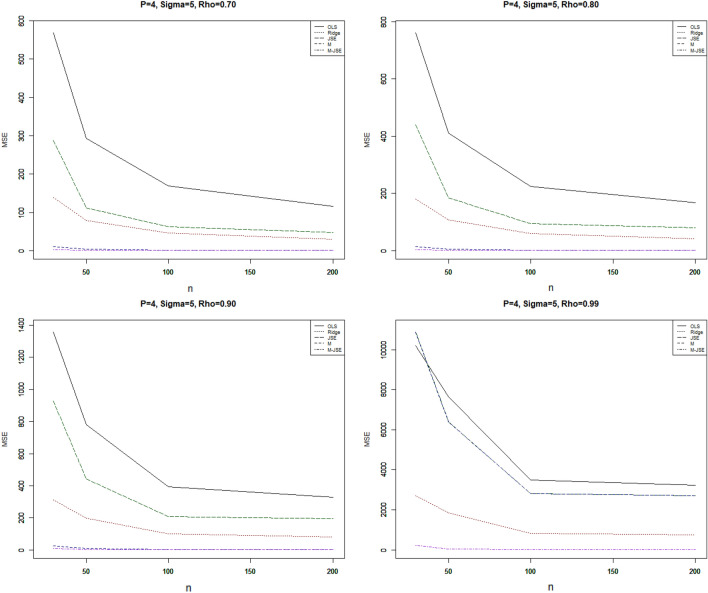
MSE vs sample size**,** For p = 4, σ = 5, 20% outliers and different ρ.

**Figure 3:**
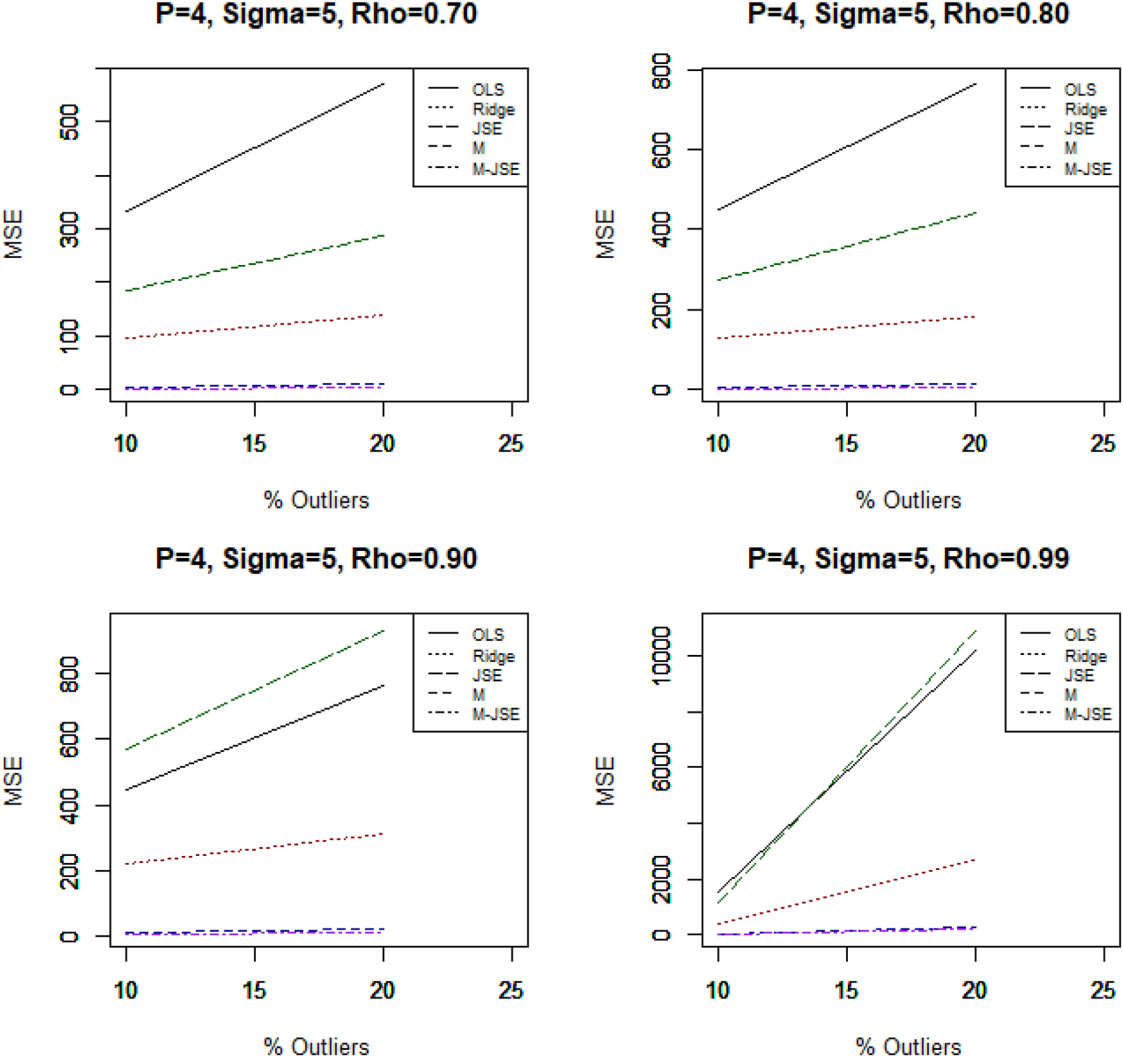
MSE vs outliers, for n = 30, p = 4, σ = 5, different values of ρ.

For a clear visualization of the simulated MSE values, we plotted MSE values vs sample size in Fig. 1 for ${p}^{*}$=4, σ = 5, 10% outliers and different ρ; in Fig. 2 for p = 4, σ = 5, 20% outliers and different ρ. The MSE values vs outliers are plotted in Fig. 3 for n = 30, ${p}^{*}$=4, σ = 5 and different ρ.

### Simulation results discussions

Our conclusions are derived from the comprehensive review of the simulation results presented in Tables 1, 2, 3, 4, 5 and 6 and Figs. 1, 2 and 3. The key findings are outlined below:

In a comprehensive evaluation, it is evident that the proposed estimator consistently outperforms OLS in all scenarios, yielding a significantly lower Mean Squared Error (MSE) value. Additionally, all of the estimators exhibit monotonic behaviors in accordance with the MSE, meaning that the estimated MSE values drop as the sample size grows. The statistics clearly show that increasing the sample size has a beneficial impact on the effectiveness of all estimators, including OLS.

The proposed estimator ${\widehat{\alpha }}_{M-JSE}$ consistently exhibits the lowest MSE values across all simulation settings, surpassing both the OLS estimator and other biased estimators. To investigate the impact of outliers on the estimated regression parameters, we considered two different percentages of outliers in the y-direction. As the percentage increases from 10 to 20%, the MSE of all estimators shows a corresponding increase. In order to assess the influence of multicollinearity on the regression parameter estimates, we varied the correlation coefficients between explanatory variables (ρ = 0.7, 0.8, 0.9, 0.99). It was observed that increasing the correlation between explanatory variables resulted in higher MSE values for all estimators. When evaluating the performance of the estimators relative to the sample size (n = 30, 50, 100, 200) while keeping p, the percent of outliers, and σ fixed, a noticeable trend emerged: the MSE consistently decreased as the sample size grew. Additionally, the parameter σ had a significant impact on the MSE, as its increase led to a corresponding rise in the MSE for all estimators. The total number of explanatory variables also influenced the MSE values for all estimators. A higher number of explanatory variables resulted in higher MSE values. Under all simulation conditions, it is observed that the proposed is the most effective choice for mitigating multicollinearity in the presence of outliers.

## Real-life application

In this section, we adopted three examples to evaluate the performance of the estimators.

### Example I

We utilized a pollution dataset that has been previously analyzed by various researchers^38,39^. The response variable is the total age-adjusted mortality rate per 100,000, which is a linear combination of 15 covariates. For a more detailed description of the data, refer to^38,39^.

**Figure 4:**
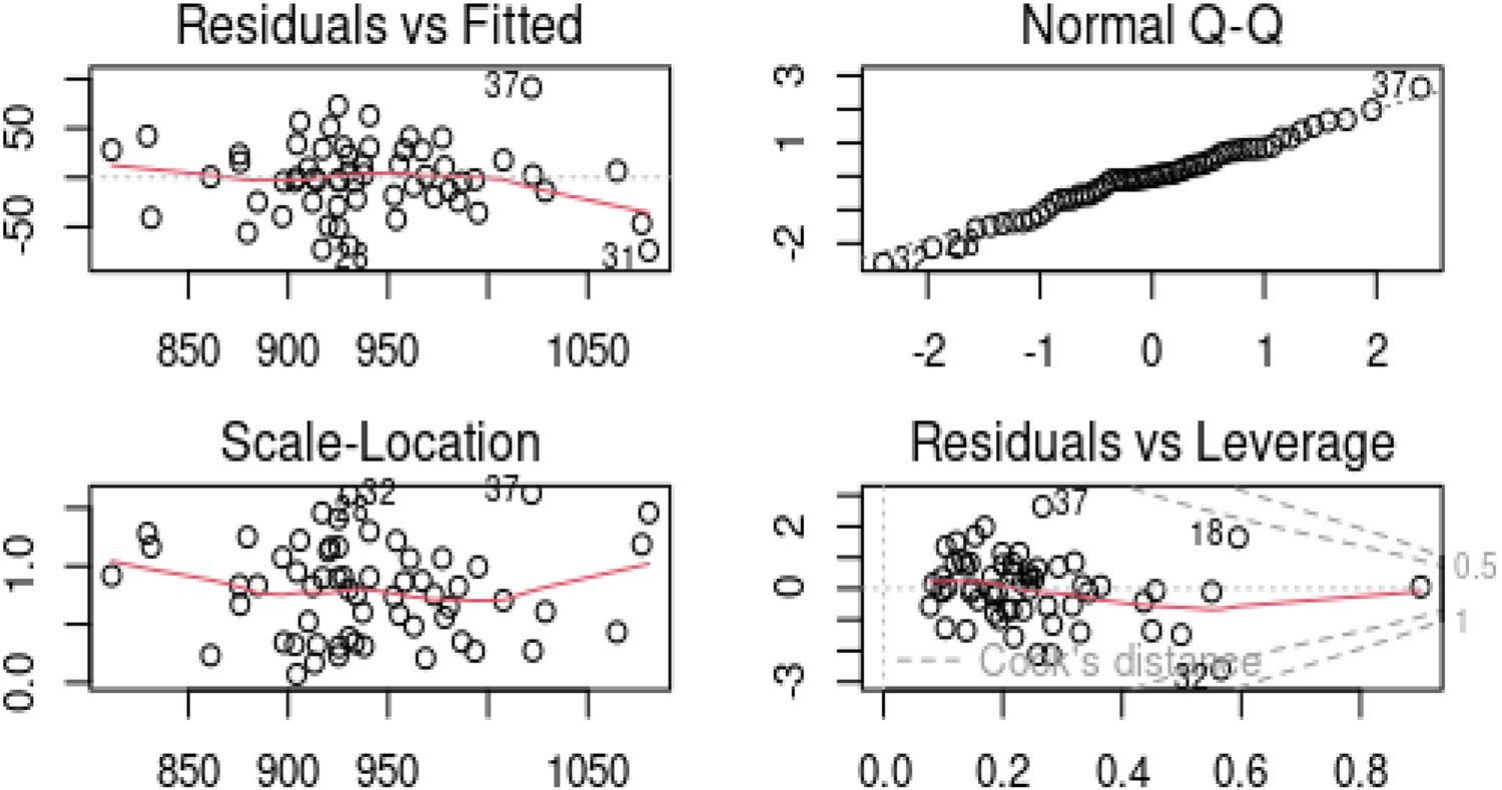
Graphical detection of outliers using pollution data.

First, we employed the least squares method to fit model (1.1) and obtained the residuals. The diagnostic plots in Fig. 4 were obtained via the residuals, which indicated that certain observations were outliers. Specifically, the residual versus fitted plot identified data points 26, 31, and 37 as outliers, and the normal Q-Q plot indicated that data points 26, 32, and 37 were outliers. The residual versus leverage plot identified observations 18, 32, and 37 as outliers, while the scale-location plot picked observations 32 and 37. These observations reveal that there are outliers in the model. Additionally, the variance inflation factor for ${x}_{i12}$ and ${x}_{i13}$ were 98.64 and 104.98, respectively, indicating a high degree of correlation between the regressors.

To address the issues of correlated regressors and outliers, we estimated the model using the ridge regression, the Stein estimator, the M-ridge, and the proposed robust Stein estimator. We compared the performance of these estimators using the scalar mean squared error (SMSE), and the regression estimates and SMSE values are provided in Table 7.

**Table 7:** Regression coefficients and SMSEs for the pollution data.

Coef	${\widehat{\alpha }}_{LS}$	${\widehat{\alpha }}_{Ridge}$	${\widehat{\alpha }}_{JSE}$	${\widehat{\alpha }}_{M-Ridge}$	${\widehat{\alpha }}_{M-JSE}$
${x}_{1}$	1.175	1.459	1.175	1.579	1.349
${x}_{2}$	− 1.516	− 2.985	− 1.516	− 2.578	− 1.342
${x}_{3}$	1.319	2.895	1.319	2.344	1.013
${x}_{4}$	11.184	6.302	11.183	6.952	11.095
${x}_{5}$	128.036	45.864	128.034	45.396	113.697
${x}_{6}$	− 1.463	− 2.593	− 1.463	4.025	5.825
${x}_{7}$	1.221	3.041	1.221	3.169	1.600
${x}_{8}$	0.007	0.007	0.007	0.008	0.008
${x}_{9}$	4.130	3.810	4.130	3.666	3.937
${x}_{10}$	0.447	0.300	0.447	− 0.681	− 0.647
${x}_{11}$	1.886	4.875	1.886	5.528	3.044
${x}_{12}$	− 0.373	− 0.401	− 0.373	− 0.235	− 0.203
${x}_{13}$	0.874	1.015	0.874	0.595	0.455
${x}_{14}$	0.160	0.145	0.160	0.215	0.232
${x}_{15}$	1.915	3.075	1.915	2.536	1.549
SMSE	2244.130	455.991	9.081	348.991	8.194

From Table 7, we observed that due to the sensitivity of the OLS estimator to correlated regressors (multicollinearity) and outliers, it exhibited the worst performance in terms of SMSE. The coefficients of all the estimates were similar, except for ${x}_{6 ,}$ where only M-ridge and M-Stein had a positive coefficient. As expected, the robust ridge dominated the ridge estimator since the ridge estimator is sensitive to outliers. However, the Stein estimator performed better than the ridge estimator, as reported in the literature. Most notably, the proposed robust version of the Stein estimator (M-JSE) outperformed every estimator under the study.

### Example II

**Figure 5:**
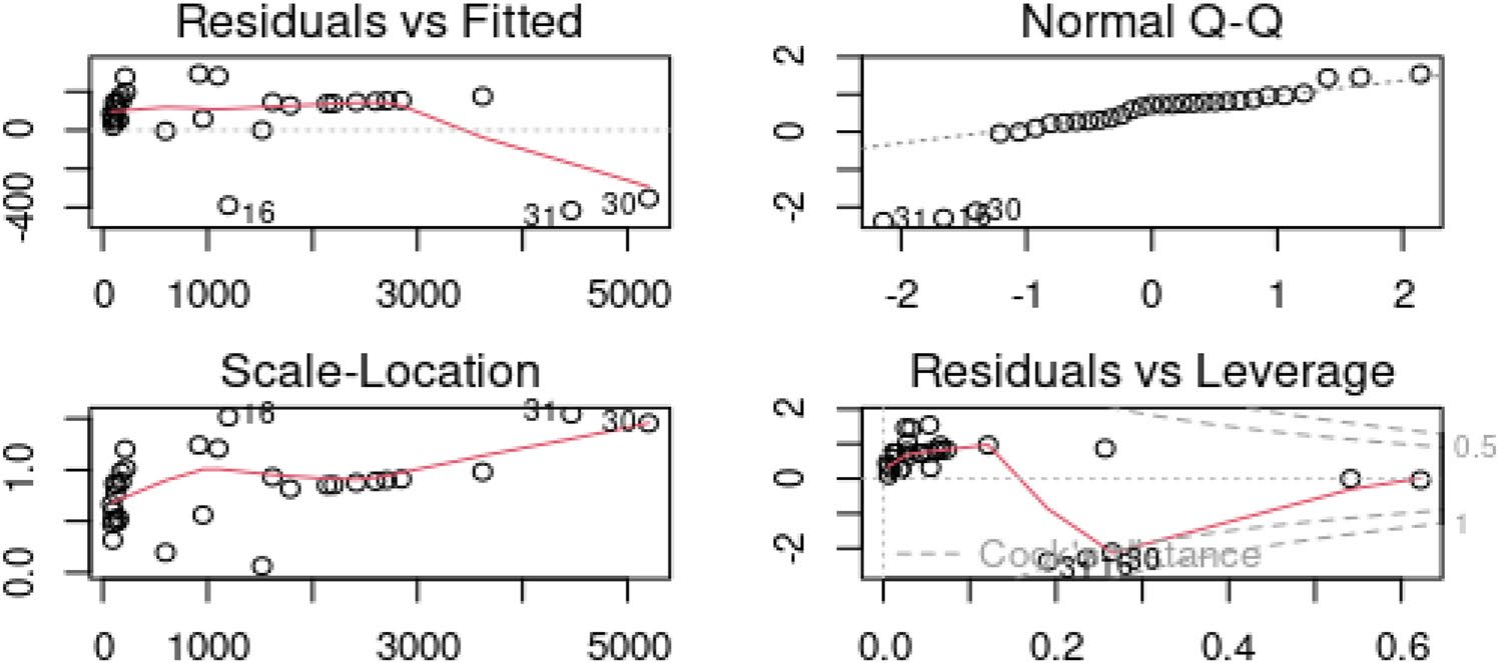
Graphical detection of outliers using import data.

The dataset was used to predict the value of a product in the manufacturing sector, based on three predictors: the value of imported intermediate (${x}_{1}$), Imported capital commodities (${x}_{2}$) and the value of imported raw materials $\left({x}_{3}\right)$^14,40,41^. A linear regression model was fitted, and the variance inflation factors were computed for each predictor, resulting in values of 128.26, 103.43, and 70.87, respectively, indicating high correlation between the predictor variables. The residual plot in Fig. 5 revealed the presence of outliers in the dataset. Outliers were identified by both the residual plot against the fitted values and the scale-location plot, which detected observations 16, 30, and 31 as outliers. The Normal Q-Q plot and Residual versus Leverage plot identified observations 31 and 30 as outliers. The residual versus leverage plot also detected observations 18, 32, and 37 as outliers, while the scale-location plot picked observation 32 and 37 as outliers. These findings indicate that the model contains both correlated regressors and outliers. The model was analyzed using several estimators, and the results were summarized in Table 8. It was observed that the regression estimate of the Stein estimator was the same as that of OLS, with a computed value of c approximately equal to 1 (c = 0.9996761). However, the Stein estimator exhibited a lower mean squared error than the OLS estimator. The ridge estimator dominated the Stein estimator in this instance, but the M-Ridge outperformed the ridge estimator by accounting for both multicollinearity and outliers. The proposed M-JSE performed the best in terms of smaller MSE.

**Table 8:** Regression coefficients and SMSE for the import data.

Coef	${\widehat{\alpha }}_{LS}$	${\widehat{\alpha }}_{Ridge}$	${\widehat{\alpha }}_{JSE}$	${\widehat{\alpha }}_{M-Ridge}$	${\widehat{\alpha }}_{M-JSE}$
${x}_{1}$	2.337	1.878	2.337	2.362	2.523
${x}_{2}$	0.573	0.617	0.573	0.518	0.511
${x}_{3}$	− 1.515	− 0.348	− 1.515	− 0.852	− 1.317
SMSE	4.723	1.837	1.355	0.793	0.690

### Example III

**Figure 6:**
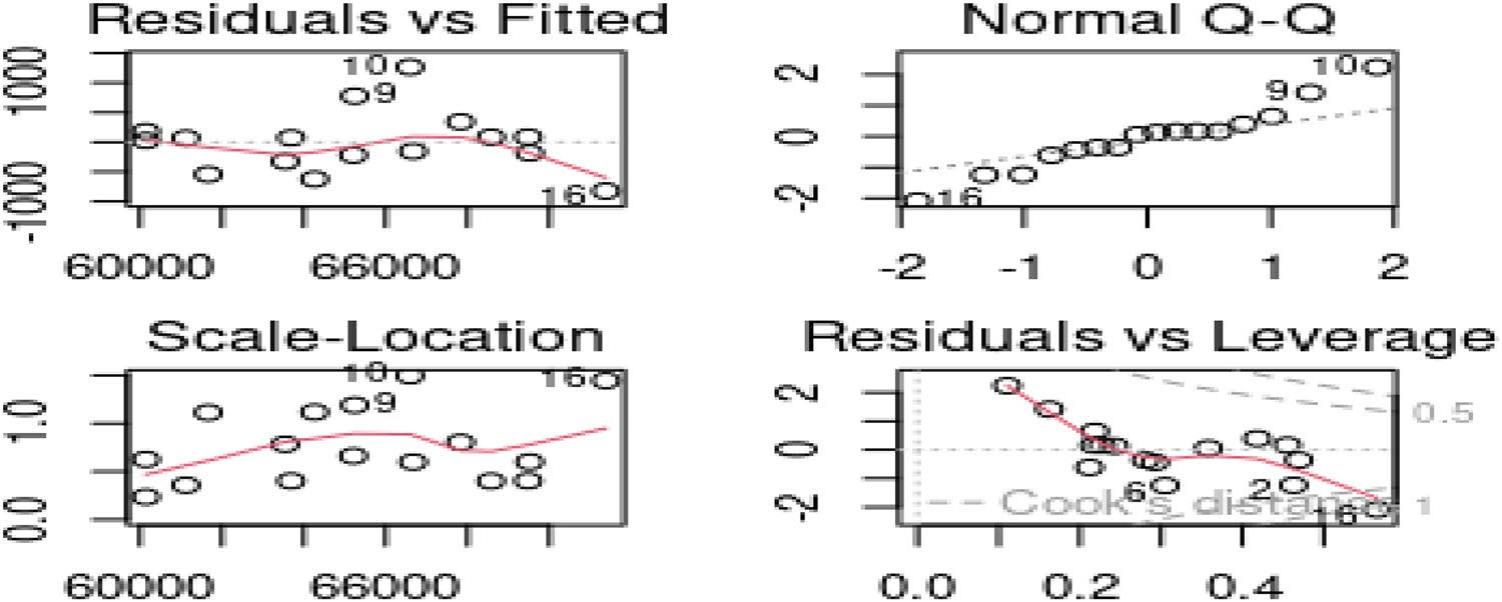
Graphical detection of outliers using longley data.

We analyzed the Longley data to predict the total derived employment, which is a linear function of the following predictors: gross national product implicit price deflator, gross national product, unemployment, size of armed forces, and non-institutional population 14 years of age and over^33,38–40,42,43^. The literature indicates that the model suffers from multicollinearity. Additionally, Fig. 6 shows that certain observations are anomalous, namely data points 9, 10, and 16.

We used both robust and non-robust estimators to analyze the data, and the results are presented in Table 9. The table indicates that the regression estimates of OLS and Stein are the same, with a value of c = 1. However, the Stein estimator has a lower SMSE than OLS. The Stein estimator dominates the ridge and robust ridge estimators in this instance. Furthermore, the proposed robust Stein estimator provides optimal performance based on the results.

**Table 9:** Regression coefficients and MSEs for the pollution data.

Coef	${\widehat{\alpha }}_{LS}$	${\widehat{\alpha }}_{Ridge}$	${\widehat{\alpha }}_{JSE}$	${\widehat{\alpha }}_{M-Ridge}$	${\widehat{\alpha }}_{M-JSE}$
${x}_{1}$	217.217	99.388	217.217	144.229	180.799
${x}_{2}$	− 0.010	− 0.004	− 0.010	− 0.006	− 0.008
${x}_{3}$	0.453	0.527	0.453	0.498	0.475
${x}_{4}$	− 1.396	− 1.335	− 1.396	− 1.324	− 1.343
${x}_{5}$	− 0.579	− 0.409	− 0.579	− 0.540	− 0.593
SMSE	11,188.401	2342.558	1.276	1055.014	1.030

In summary, the Longley data analysis indicates that the model suffers from multicollinearity and contains anomalous observations. However, using the robust Stein estimator provides the best performance among the estimators considered in this study.

## Some concluding remarks

Linear regression models (LRMs) are widely used for predicting the response variable based on a combination of regressors. However, correlated regressors can decrease the efficiency of the ordinary least square method. Alternative methods such as the Stein and the ridge estimators can provide better estimations in such situations. However, these methods can be sensitive to outlying observations, leading to unstable predictions.

To address this issue, researchers have previously combined the ridge estimator with robust estimators (such as M-estimators) to account for both correlated regressors and outliers.

In this study, we developed a new biased estimator that offers an alternate approach to handling multicollinearity in linear regression, it is boosted Stein estimator by combining the M-estimator with the Stein estimator. Pseudo random numbers are created for both the independent and dependent variables in a Monte Carlo experiment. Different sample sizes, correlation strengths, and quantities of independent variables are taken into account. Our simulation and application results demonstrate that the robust Stein estimator outperforms the other estimators considered.

It is noted that, in the case of high multicollinearity, the suggested estimator showed its best performance by means of the reduction of the estimated MSE values and it is not affected by multicollinearity as much as other estimators. According to the tables, there is some difference between the performances of the suggested estimators according to the shrinkage parameter that is used and it may be concluded that, ${k}_{m}$ is the best shrinkage parameter among others in most cases.

The findings of this paper will be beneficial for practitioners who encounter the challenge of dealing with multicollinearity and outliers in their data. By using the Robust Stein estimator, they can obtain more stable and accurate predictions.

While this study has made substantial progress in addressing the challenges of LRMs, there are still avenues for further exploration. Future research endeavors should consider incorporating other robust estimators including the robust Liu estimator, Robust Liu-type estimator, robust linearized ridge estimator, Jackknife Kibria-Lukman M-Estimator, Modified Ridge-Type M-Estimator to conduct a more comprehensive comparative analysis^13,14,45–47^. This will contribute to a deeper understanding of the strengths and limitations of different approaches in handling complex data scenarios.

Another potential direction for future research is the extension of the current study using neutrosophic statistics. Neutrosophic statistics is an extension of classical statistics that is particularly useful when dealing with data from complex processes or uncertain environments^48–53^. By incorporating neutrosophic statistics, we can account for additional sources of uncertainty and variability, which may further enhance the robustness and applicability of our proposed estimator.

## Supplementary Information

Supplementary Information 1.

Supplementary Information 2.

Supplementary Information 3.

## Data Availability

All data analysed during this study are included as Supplementary Files.
